# The pathways from parental and neighbourhood socioeconomic status to adolescent educational attainment: An examination of the role of cognitive ability, teacher assessment, and educational expectations

**DOI:** 10.1371/journal.pone.0216803

**Published:** 2019-05-22

**Authors:** Dominic Weinberg, Gonneke W. J. M. Stevens, Catrin Finkenauer, Bert Brunekreef, Henriëtte A. Smit, Alet H. Wijga

**Affiliations:** 1 Department of Interdisciplinary Social Science, Faculty of Social and Behavioural Sciences, Utrecht University, Utrecht, The Netherlands; 2 Department of Biological Psychology, Vrije Universiteit Amsterdam, Amsterdam, The Netherlands; 3 Institute for Risk Assessment Sciences (IRAS), Division of Environmental Epidemiology, Utrecht University, Utrecht, The Netherlands; 4 Julius Center for Health Sciences and Primary Care, University Medical Center Utrecht, Utrecht, The Netherlands; 5 Centre for Prevention and Health Services Research, National Institute for Public Health and the Environment (RIVM), Bilthoven, The Netherlands; University of Edinburgh, UNITED KINGDOM

## Abstract

Adolescents with high educational attainment generally have better outcomes across the lifespan than adolescents with lower educational attainment. This study investigated how three measures of socioeconomic status (SES)–maternal education, paternal education, and neighbourhood SES–combined to predict adolescent educational attainment (track level at age 17). We proposed three mechanisms for this pathway: cognitive ability (at age 11), primary school teacher assessment (stating the secondary education level suitable for a child at age 11), and educational expectations (at age 14). Using the data of 2,814 Dutch adolescents from the Prevention and Incidence of Asthma and Mite Allergy (PIAMA) study, logistic regressions tested associations between SES and educational attainment. Structural equation modelling (SEM) tested mediational pathways between SES and educational attainment. In models with three SES measures, having a medium-educated mother was associated with higher educational attainment relative to having a low-educated mother (OR; 95% CI: 1.83; 1.41–2.38), and having a high-educated mother was associated with higher educational attainment relative to having a low-educated mother (OR; 95% CI: 3.44; 2.59–4.55). The odds ratios for paternal education showed a similar pattern. We found no association between neighbourhood SES and adolescent educational attainment, so neighbourhood SES was removed from further analyses. Mediational analyses revealed that cognitive ability (30.0%), teacher assessment (28.5%), and educational expectations (1.2%) explained 59.8% of the total association between parental SES and educational attainment. The results showed that mother education and father education were both important for understanding the strong association between parental SES and adolescent educational attainment. In the Netherlands, the association between parental SES and educational attainment can be largely explained by cognitive ability and teacher assessments.

## Introduction

Educational attainment is a key goal of adolescence [1]. High educational attainment is associated with, and expected to have a causal effect on, positive outcomes later in life through many economic, health-behavioural, and social-psychological mechanisms. These outcomes include getting a stable, well-paid, high-status job, healthier behaviour and longer life expectancy, and increased political engagement and trust [2–6]. In sharp contrast, adolescents with low educational attainment are more likely than those with high attainment to end up in cycles of poverty, ill-health, and deprivation [6–8]. Processes of globalisation, technological development, and labour market polarisation further increase the importance of educational attainment for outcomes throughout life [9,10]; highly educated citizens can best take advantage of these processes [11]. It is therefore important to better understand the predictors and pathways which are related to educational attainment during adolescence.

An ‘ecological’ perspective on development emphasises the interactions between individuals and the familial, community and societal contexts they are embedded within [12–14]. The perspective provides a theoretical framework for understanding how different levels of the social context are related to adolescent educational attainment [15,16]. Socioeconomic status (SES), which comprises material and social resources and assets, as well as status in the social hierarchy, is a central component of the social context [17]. To better understand how SES is related to adolescent educational attainment, it is important to not only consider different contextual indicators of SES, but also investigate pathways which may explain the SES-attainment association.

### SES and educational attainment

Parental SES is strongly and persistently linked to adolescent educational attainment: on average, high-SES parents have children with higher educational levels than low- or medium-SES parents [18–20]. The relation between parental SES and educational attainment, described by sociologists as ‘social reproduction’ [21], and by economists as the ‘intergenerational transmission of human capital’ [22], contributes to limited social mobility between generations. Parental education is considered a stable measure of parental SES, because it is established at an early age and is fairly constant over time [23].

Evidence from recent decades across many countries has shown that both parents’ education levels are similarly predictive of adolescent educational attainment, though the relative importance of the mother and the father may depend on who has the highest education level [24,25]. Assortative mating, whereby highly educated individuals are more likely to also have highly educated partners, may further limit social mobility between generations [26]. Changes in educational levels, especially among women, with consequences for assortative mating, suggest that current research on the impact of parental education on adolescent educational attainment should include the education levels of both parents [27,28].

As they grow older, adolescents increasingly spend time outside the home in their neighbourhoods and are influenced by the social space where many of their interactions occur [29]. Neighbourhoods may vary in their social cohesion, physical surroundings, and the level of resources provided by public services, all of which are related to adolescent development and consequently educational attainment [30,31]. As such, adolescent outcomes may not only be related to their parental SES, but also to the SES of their neighbourhood. Indeed, adolescents who grow up in a neighbourhood where the population has a low average SES (i.e., low neighbourhood SES) have lower educational attainment, and living for longer in higher SES neighbourhoods during childhood and adolescence is related to higher educational attainment [32–34].

Many studies have found that the relation between neighbourhood SES and adolescent achievement outcomes holds over and above the relation between parental education and adolescent achievement, but less is known about how parental education and neighbourhood SES interact [35]. One study in England found that the negative relationship between living in low SES neighbourhoods and educational attainment was stronger for adolescents with high-educated parents than for adolescents with low-educated parents [36]. Another study in the UK found that adolescents with low-educated parents, neighbourhood SES was a more important predictor than parental SES for adolescent educational attainment [37]. In light of these differing findings, we sought to explore the interaction between parental and neighbourhood SES on educational attainment in adolescence.

### Pathways from SES to educational attainment

Although the link between SES and educational attainment is well-established, questions remain about which mechanisms, related to parental and neighbourhood SES, predict adolescent educational outcomes. The ecological perspective emphasises that SES may be associated with adolescent development through several levels: adolescents’ individual characteristics, their developing agency, and contextual processes [12,16]. The principal individual mechanism by which SES may be related to educational attainment is adolescent cognitive ability [38,39]. Parental education is strongly positively associated with adolescent cognitive ability: both are related to parental cognitive ability and they share genetic and environmental influences [40,41]. Neighbourhood SES also shows associations with adolescent cognitive ability, possibly because of poorer quality teaching, fewer resources, less challenging curricula, or the inaccessibility of learning materials in low compared to high SES neighbourhoods, though these mechanisms may be stronger in the USA than in Europe [35,42–44]. Adolescent cognitive ability is positively associated with educational attainment [39,45]. Thus, we expected that cognitive ability would mediate the association between SES and educational attainment.

The ecological perspective also emphasises that schools and teachers are a particularly important context for development [12,13]. Therefore, a second mechanism by which parental and neighbourhood SES may be associated with adolescent educational attainment is through teacher assessment of adolescent capabilities. Social reproduction theory suggests that higher educated parents transmit to their children not only their genes but also more cultural capital–status-based cultural signals, such as attitudes, preferences and behaviours [21,46]. This gives children of higher educated parents ways of interacting with peers and adults that teachers judge positively [46]. Additionally, higher educated parents have the resources and knowledge to effectively advocate for teachers to support the academic ambitions of their children, who may then make more positive assessments of the capabilities of those adolescents than is justified by evidence of their cognitive ability alone [47,48]. In contrast, teachers have lower than justified assessments of the educational potential of adolescents of lower educated parents [49]. Parental education level may also be associated with non-cognitive traits such as personality, which may be genetically transmitted to children and subsequently influence teacher assessments [50]. In addition, neighbourhood SES may also be related to teacher assessment of adolescent capabilities. By definition, low-SES neighbourhoods contain a relative concentration of students with lower educated parents and this concentration is usually reflected in the school population, in turn negatively affecting the expectations of teachers for all students [51,52]. Evidence from the Netherlands has shown that teachers had higher educational assessments for students in classes where there were fewer students from low-SES families [53]. Teacher assessment of educational capabilities is thus expected to be an important second mediator of the association between SES and educational attainment in the Netherlands because of its impact on the education track adolescents can follow [54].

A third mechanism by which parental education and neighbourhood SES may be associated with adolescent educational attainment is through adolescent educational expectations. Adolescents' visions of their future career and lifestyle are an important part of their identity development and are important for the choices adolescents make in life [55,56]. Parents may act as ‘expectancy socializers’, encouraging and moulding their children’s own expectations [57]. Several studies have found that higher educated parents have children with more ambitious educational expectations (for a review, see [58]). Neighbourhood SES may also be related to adolescent educational expectations, because adolescents typically feel a sense of belonging to, and pride in, their neighbourhood and thus their attitudes and beliefs develop in interaction with people living nearby [52,59,60]. In higher SES neighbourhoods, higher levels of people in employment can encourage adolescents to be school-focused [61]. In low SES neighbourhoods, there is greater heterogeneity in educational expectations and adolescents may be influenced by neighbours with either educational or alternative goals [33]. Thus, adolescents from low-SES neighbourhoods are expected to have lower educational expectations than their peers in high-SES neighbourhoods. Educational expectations are related to adolescent educational attainment, possibly through choices, decisions, and activities [16,62]. Thus, our final mediational hypothesis was that educational expectations would also mediate the association between SES and educational attainment.

Evidence suggests that all three mediational pathways–cognitive ability, teacher assessment, and educational expectations–at least partly explain the relation between parental education and neighbourhood SES and educational attainment, but to our knowledge no studies have tested these three mediators in one model. There is evidence that all three mediators included in the study are related to each other [52,63,64]. These associations suggest that the effects of each proposed mediator may attenuate once other pathways are included. By examining these mediators together in one model, we will shed light on the relative importance of each mediator in explaining the relation between SES and educational attainment.

### The present study: Pathways from SES to educational attainment in the Netherlands

Using data from a large prospective study in the Netherlands, this study first investigated whether parental SES, comprising maternal education and paternal education as separate indicators, and neighbourhood SES predicted adolescent educational attainment. We took a stepwise approach: initially modelling the effect of these factors individually, then including all three effects simultaneously, then adding interactions between them. We expected significant effects for all three measures of SES, even after controlling for the other measures [65].

Secondly, we analysed three mechanisms expected to explain the pathway from SES to educational attainment: cognitive ability, teacher assessment, and educational expectations. For mediation analysis, we combined maternal education, paternal education, and neighbourhood SES into one latent SES measure [66]. Based on research in other countries, we expected that higher SES would predict greater cognitive ability, higher teacher assessment, and higher educational expectations, and these, in turn, would predict higher educational attainment in adolescents [16,67]. We analysed the effects of the three proposed mediators individually and then in combination to assess whether they continued to play a role once other possible mechanisms were considered.

This study took place in the Netherlands, where the education system is characterised by its strong and early stratification, with academic tracking into educational trajectories at age 12 [68,69]. In theory, parents and children are free to choose the secondary school (although some schools need to do a lottery whenever they have too many student applications) and schools are free to select any students. However, in practice, most secondary schools consider two pieces of information at the end of primary education as an entry requirement, which they can weigh as they choose: scores on a national (Cito) school leaver test and a teacher assessment of the appropriate school level [53].

The teacher assessment, provided by the main teacher of the child’s primary school, is based on cognitive ability (e.g., Cito Test score), non-cognitive abilities (i.e., an evaluation of children’s motivation, characteristics, and capacities), and judgements about their home situation [49,70,71]. According to a survey of schools by the Dutch Inspectorate of Education, when giving assessments, more than three-quarters of schools take into account the home situation, such as how stable it is and the extent of parental support for their child’s education [72]. The teacher's assessment ranges through six educational tracks from lowest to the highest, two of which are ‘bridge class’ assessments. In bridge classes, students spend the first year or two of secondary education in mixed classes (i.e., a combination of two educational levels) which could lead to either of two adjacent levels. The teacher's assessment often corresponds with the secondary school level indicated by the Cito Test [73]. Yet, in roughly 35% of cases, when teachers believe that students are better off in a track other than the one indicated by the Cito Test, they give alternative assessments [72]. Mobility between tracks is possible during secondary education: national registry-based data from 2012/13 (when our participants were 17/18) showed that, compared to the teacher assessment: 16% of adolescents finished in a lower track; 16% in the lower of two bridge class tracks; 45% in the corresponding track; 14% in the higher of two bridge class tracks; and 10% in a higher track [74]. Despite relatively low levels of inequality in the Netherlands, there is evidence that inequity is growing, perhaps due to the Great Recession [68, 75,76]. The relation between parental education and neighbourhood SES with adolescent educational attainment may be relatively strong in the Netherlands; decisions about educational tracking are made early in the students’ school career, when less is known about their academic potential in comparison with parental SES [68,69,77].

## Method

### Participants

The Medical Ethical Committees of the participating institutes approved the study (Rotterdam, start project MEC 132.636/1994/39 and 137.326/1994/130; Groningen, start project MEC 94/08/92; Utrecht, start project MEC-TNO judgement 95/50; Utrecht, age 4 years CCMO P000777C; Utrecht, age 8 years CCMO P04.0071C, protocol number 04-101/K; Rotterdam, age 8 years MEC 2004–152; Groningen, age 8 years M 4.019912; Utrecht, age 12 years METC protocol number 07-337/K). Parents, carers or guardians gave written informed consent on behalf of all the minors/children involved in the study [78].

Data on Dutch adolescents were obtained from the Prevention and Incidence of Asthma and Mite Allergy (PIAMA) study, originally designed to investigate the influence of lifestyle and environment on the development of asthma, allergy, and lung function in children and adolescents [78]. The study recruited pregnant women from the general population in three parts of the Netherlands (North: provinces Groningen, Friesland, and Drenthe; Central: Utrecht and Gelderland; West: Rotterdam and surrounding municipalities). Their children (*N* = 3,963), born in 1996/1997, have been followed from birth onwards. Data were collected across several waves, including yearly from 1 to 8 years of age and at the age of 11, 14, and 17, using questionnaires on family characteristics, lifestyle, physical, and psychosocial health. The PIAMA study population, in comparison with the Dutch population at the time, was more highly educated (35% compared to 22%) and had fewer non-Western immigrant parents (3.6% compared to 15%), which reflects the fact that higher educated native speakers are more likely to participate in research involving lengthy questionnaires [78]. The study population was similar to the general population with respect to maternal age at childbirth.

Analyses were based on participants for whom both maternal education and paternal education were assessed when the child was 1 year old, and at least one further (mediating or outcome) measure was available (*N* = 2,814). Compared to the original PIAMA study population, the analysis sample under-represented adolescents with low SES (*Χ*^*2*^_*Mother education*_ (2, *N* = 3807) = 115.32, *p* < .001; *Χ*^*2*^_*Father education*_ (2, *N* = 3,761) = 75.10, *p* < .001); and *t*_Neighbourhood SES_ (3907) = 6.91, *p* = .045). Missing data were modelled with the WLSMV estimator (see below), allowing for missing observations instead of pairwise or listwise removal of participants. Sensitivity analysis using multiple imputation revealed that any bias due to study dropout related to participant characteristics (such as parental education) did not have a substantial effect on the findings.

### Measures

#### Maternal education and paternal education

Information about the highest level of education attained by the mother and the father was obtained when their children were roughly 1 year old, and was categorised as low (primary school, lower vocational, or lower secondary education), medium (intermediate vocational education or intermediate/higher secondary education), or high (higher vocational education and university), in accordance with prior research (e.g., [70]). In the Netherlands, education correlates strongly with other indicators of SES (e.g., one study found a correlation of .49 with income [46]).

#### Neighbourhood SES

Information on neighbourhood socioeconomic status (SES) was obtained from The Netherlands Institute for Social Research (SCP; [79]). The SCP’s indicator of neighbourhood-level (four-digit postal code) SES represents the educational, occupational, and economic status of the neighbourhood (i.e., average income, the percentage of people with a low income, the percentage of low-educated people and the percentage of people who do not work) and was collected in 2006. Each participants’ score was based on their reported postal code in 2006, when children were aged 9 or 10.

#### Educational attainment

Adolescents reported their education level at age 17, with responses coded ordinally: 1 = VMBO/MBO (vocational education track); 2 = HAVO/HBO (general secondary education track); 3 = VWO/University (university education track). Students in other education were coded as missing (e.g., studying abroad, *n* = 14).

#### Cognitive ability

In the February of their final year of primary education (i.e., aged 11–12), most Dutch students take the Cito Eindtoets Basisonderwijs (“Cito Final Test Primary Education”), designed to assess cognitive ability [68]. The Cito Test is a good instrument for assessing individual differences in cognitive ability, with scores ranging from 501 to 550, and it provides guidance for the appropriate secondary educational level. Parents were sent a short questionnaire asking about the overall standardised Cito Test score and the teacher's assessment of the child's educational abilities (see below; [73]). The Cito Test is a valid instrument for assessing individual differences in cognitive ability: research finds that the Cito score correlates with IQ at age 12 (*r* = 0.63) [80]. The test comprises 240 multiple choice items assessing language, mathematics, information processing, and world orientation [81].

#### Teacher assessment

The primary school teacher's assessment is provided in the spring of the final year of primary education, once teachers know the results of the Cito Test. The assessment states the secondary education level(s) suitable for a child according to the teacher. We coded this variable (based on the highest level stated by the teacher): 1 = preparing for labour market (VMBO-lbk); 2 = preparing for vocational education (VMBO-gt); 3 = combination class of preparing for vocational education and secondary general education (VMBO/HAVO); 4 = preparing for secondary general education (HAVO); 5 = combination class of preparing for secondary general or pre-university education (HAVO/VWO); 6 = preparing for university education (VWO). In 3 cases teachers gave an assessment of “VMBO”, which was coded 1.

#### Educational expectations

Adolescents reported their educational expectations at age 14 in response to the following question: “What are your plans after this education?” Based on the extent to which they indicated expectations for further educational attainment, the responses were coded ordinally: 0 = “I don’t know yet”; 1 = “I'm going to work”; 2 = “I'm going to attend a course where I partly work and partly go to school”; 3 = “I will start with further or other education or study”.

### Data analysis

To test the independent associations between maternal education, paternal education and neighbourhood SES with adolescent educational attainment, we ran ordinal logistic regressions in SPSS (version 24) based on the three ordered educational attainment categories. We fitted a logit model, entering maternal education and paternal education into the regressions as ordinal predictors (Models 1a and 1b) and neighbourhood SES as a continuous predictor (Model 1c). Next, a regression was run with all the predictors added simultaneously (Model 1d). Interaction terms (i.e., maternal education x paternal education, maternal education x neighbourhood SES, paternal education x neighbourhood SES, and maternal education x paternal education x neighbourhood SES) were added to the final model (Model 1e).

To test mediational pathways between the SES measures and educational attainment, relationships between the variables were examined with structural equation modelling (SEM), using Mplus (Version 8; [82]). Models were estimated using weighted least squares mean and variance adjusted estimation (WLSMV), which is suited to categorical data [83]. Following recommendations to reduce variance in complex models to improve model convergence and identification we centred cognitive ability scores and divided them by 10 [82]. We bootstrapped effects 2,000 times with bias-corrected standard errors and used theta parameterisation due to the presence of categorical predictors [82,84]. Goodness-of-fit was evaluated using two indices, with excellent model fit indicated by CFI ≥ .95 and RMSEA < .05 [85].

We conceptualised SES as caused by parental education and neighbourhood SES, so our SES variable was modelled as a formative indicator (i.e., a weighted combination of the parental education and neighbourhood SES variables, which reduces measurement error [86,87]). Adding an interaction term between maternal education and paternal education to Model 2 made no difference to the results and was therefore removed to make the model more parsimonious [88]. Models 2a-c successively included paths for the three hypothesised mediators (cognitive ability, teacher assessment, and educational expectations) between the SES indicator and educational attainment, as well as including a direct path from SES to educational attainment. Model 2d tested all three mediators concurrently, with these variables allowed to correlate. Evidence for mediational pathways was established based on the Mplus estimation of indirect effects, which uses Sobel’s [89] asymptotic z test. A summary of the models can be found in Table 1.

**Table 1:** Summary of models.

Model	Model type	Variables included
1a	Logistic regression	Maternal education → Educational attainment
1b	Logistic regression	Paternal education → Educational attainment
1c	Logistic regression	Neighbourhood SES → Educational attainment
1d	Logistic regression	Maternal education → Educational attainment Paternal education → Educational attainment Neighbourhood SES → Educational attainment (predictors added simultaneously)
1e	Logistic regression	Maternal education → Educational attainment Paternal education → Educational attainment Neighbourhood SES → Educational attainment Interaction terms → Educational attainment (predictors added simultaneously)
2a	Structural equation model	SES → Cognitive ability Cognitive ability → Educational attainment SES → Educational attainment
2b	Structural equation model	SES → Teacher assessment Teacher assessment → Educational attainment SES → Educational attainment
2c	Structural equation model	SES → Educational expectations Educational expectations → Educational attainment SES → Educational attainment
2d	Structural equation model	SES → Cognitive ability Cognitive ability → Educational attainment SES → Teacher assessment Teacher assessment → Educational attainment SES → Educational expectations Educational expectations → Educational attainment SES → Educational attainment (predictors added simultaneously)

*Note*. SES = Formative indicator of Maternal education, Paternal education, and Neighbourhood SES.

## Results

### Descriptive statistics

Table 2 gives the correlations between the observed variables: all variables were related positively to each other, except for neighbourhood SES (*M* = 0.40, *SD* = 0.81), which was not related to adolescent educational expectations or teacher assessment. Mean levels of maternal education and paternal education were similar and the variables moderately correlated (*r* = 0.50). Table 3 gives the frequencies of the categorical measures: parents were mostly medium or high educated (80.3% of mothers and 77.2% of fathers); the most common teacher assessment was ‘preparing for university education’; and most adolescents (67.8%) expected to go into further education. The educational attainment of the adolescents was equally split between the three education tracks, with one-third in the vocational education track, one-third in the general secondary education track, and one-third in the university education track. These findings indicate that adolescents in this sample were more highly educated than the average Dutch population; over 50% of adolescents in this birth cohort were in the lowest (vocational) education track at age 14 [90]. Their cognitive ability scores (*M* = 538.91, *SD* = 7.92) were also above average (535.4; [91]).

**Table 2:** Correlations between SES measures, mediators, and educational attainment.

	Variables	2	3	4	5	6	7
SES	1. Maternal education	.50**	.06**	.29**	.33**	.06**	.36**
	2. Paternal education		.13**	.29**	.35**	.06**	.39**
	3. Neighbourhood SES			.05* ^a^	.04 ^a^	.02	.05*
Mediators	4. Cognitive ability				.86** ^a^	.09**	.69**
	5. Teacher assessment					.11**	.73**
	6. Educational expectations						.13**
Outcome	7. Educational attainment						

Note.

^a^ Correlation coefficients between Neighbourhood SES, Cognitive ability, and Teacher assessment are Pearson’s (all other correlations are Spearman’s). *n*s vary due to missing data, ranging from 1,236–2,814.

* *p* < .05

** *p* < .01.

**Table 3:** Frequencies of parental education and categorical mediators.

	*%*
**Maternal education** (*n* = 2,814)	
Low	19.7
Medium	41.4
High	38.9
**Paternal education** (*n* = 2,814)	
Low	22.8
Medium	34.1
High	43.1
**Teacher assessment** (*n* = 2,096)	
Preparing for labour market	7.5
Preparing for vocational education	13.5
Combination class of preparing for vocational education and secondary general education	13.2
Preparing for secondary general education	16.4
Combination class of preparing for secondary general or pre-university education	20.1
Preparing for university education	29.3
**Educational expectations** (*n* = 2,474)	
I don’t know	22.8
Work	1.6
Partly work, partly school	7.8
Further education	67.8

### SES and adolescent educational attainment at age 17

Table 4 presents the number and proportion of adolescents in each educational track, broken down by parental education. Among adolescents with low-educated mothers, 61% of adolescents had the lowest level of educational attainment, 27% had a medium level of educational attainment and only 13% had the highest level of educational attainment. In contrast, among adolescents with high-educated mothers, only 17% had the lowest level of educational attainment, 34% had a medium level, and 49% the highest level. Adolescents with medium-educated mothers were more evenly split between the three groups: 39% had the lowest level of educational attainment, 36% had a medium level, and 25% the highest level. The proportions based on paternal education paralleled those for maternal education.

**Table 4:** Percentage of adolescents with each educational attainment level by parental education level (n = 1,978).

	*N*	*% in educational track*		
		VMBO/ MBO	HAVO/ HBO	VWO/ University
**Maternal education**				
Low	336	60.4	26.8	12.8
Medium	805	38.9	36.0	25.1
High	837	17.6	33.9	48.5
**Paternal education**				
Low	402	61.2	25.9	12.9
Medium	652	41.0	34.7	24.4
High	924	16.2	36.1	47.6
**Total**	1,978	33.5	33.6	32.9

Table 5 shows the relation between parental education and educational attainment, with low-educated parents as the reference category. The first three models (1a-1c) showed that maternal education (Wald χ2(2) = 258.37, p < .001) and paternal education (Wald χ2(2) = 298.70, p < .001), but not neighbourhood SES (Wald χ2(1) = 3.84, p = .050), were associated with adolescent educational attainment. When all three measures of SES were included together in one model, maternal education (Wald χ2(2) = 80.72, p < .001) and paternal education (Wald χ2(2) = 131.14, p < .001) remained statistically significant, and neighbourhood SES (Wald χ2(1) = 0.00, p = .965) was still not statistically significant (Model 1d). The results of this last model showed that adolescents with medium-educated mothers had nearly 2 times the odds of having higher educational attainment relative to adolescents with low-educated mothers, and adolescents with high-educated mothers had over 3 times the odds of having higher attainment relative to adolescents with low-educated mothers. The odds ratios for paternal education showed a similar pattern. Supplementary analysis showed that, in a model with all three measures of SES, when medium-educated mothers was used as a reference category, adolescents with high-educated mothers had nearly 2 times the odds of having higher educational attainment relative to adolescents with medium-educated mothers (OR = 1.88, 95% CI [1.54–2.29], p < .001). The equivalent odds ratio for adolescents with high-educated fathers relative to medium-educated fathers was slightly higher (OR = 2.36, 95% CI [1.92–2.90], p < .001). The results showed that both mother education and father education remained independently associated with higher adolescent educational attainment when the other parent’s education was included. There were no main or interaction effects for neighbourhood SES, so we included only interactions between maternal education and paternal education in our final model (Model 1e). The interaction model showed that there was no significant interaction between maternal education and paternal education (Wald χ2(4) = 4.728, p = .316).

**Table 5:** Odds ratios of higher educational attainment from logistic regression models with SES measures.

	Odds Ratio (95% Confidence Interval)			
	Model 1a (Mother)	Model 1b (Father)	Model 1c (Neighbourhood)	Model 1d (Simultaneous)
ME (Low)	1			1
ME (Medium)	**2.39 (1.86–3.06)**			**1.83 (1.41–2.38)**
ME (High)	**6.94 (5.38–8.95)**			**3.44 (2.59–4.55)**
PE (Low)		1		1
PE (Medium)		**2.31 (1.81–2.95)**		**1.81 (1.41–2.34)**
PE (High)		**7.2 (5.68–9.14)**		**4.28 (3.28–5.57)**
Neighbourhood SES			1.11 (1.00–1.23)	1.00 (0.90–1.11)

*Note*. ME = Maternal education. PE = Paternal education. Maternal education (Low) and Paternal education (Low) is the reference group. Model 1d included Maternal education, Paternal education, and Neighbourhood SES simultaneously. Bold figures indicate significant relations, where group members had a greater chance for higher educational attainment relative to the reference group.

### Pathways from SES to adolescent educational attainment at age 17

**Fig 1:**
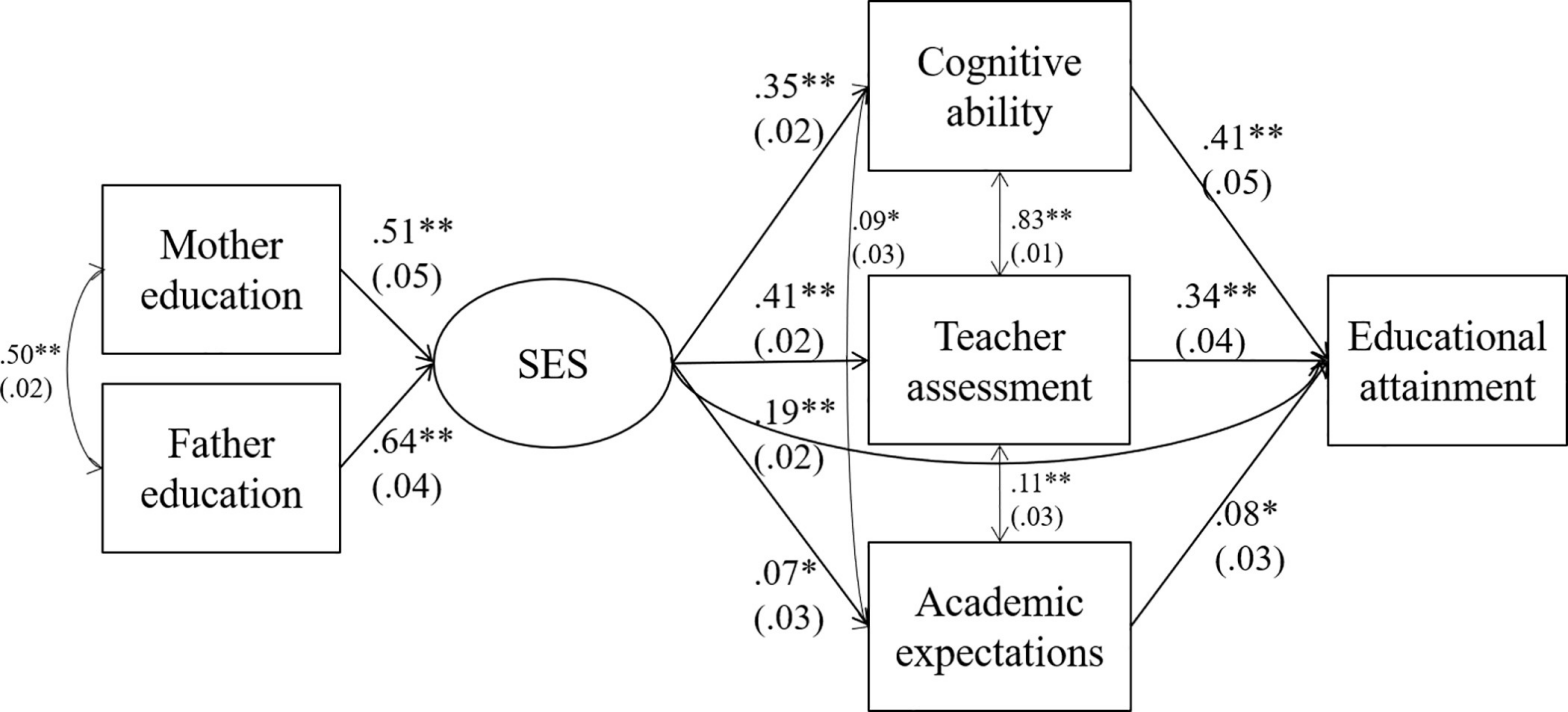
Standardised path coefficients from structural equation model 2d. * p < .01. ** p < .001.

Neighbourhood SES did not predict adolescent educational attainment at age 17, and because sensitivity analyses revealed that including Neighbourhood SES in the SES indicator made no substantial difference to subsequent models (see S1 Table), we used only maternal education and paternal education for the formative indicator for SES. Table 6 shows the results of models which tested the hypothesised mediators of the SES indicator on educational attainment. Model 2a showed that, consistent with our hypothesis, SES predicted adolescent cognitive ability at age 11, and cognitive ability at age 11, in turn, predicted educational attainment. A test of mediation found that cognitive ability mediated the relationship between SES and adolescent educational attainment at age 17, explaining 50.3% of the total association. Models 2b and 2c showed similar results for teacher assessment (at age 11) and educational expectations (at age 14); both were predicted by SES and in turn predicted adolescent educational attainment at age 17. Separate mediation tests found that each significantly mediated the path from SES to educational attainment, with teacher assessment explaining 59.7% of the total association and educational expectations 2.2% of the total association. Finally, Model 2d showed that these associations remained significant once all hypothesised mediators were included in a model concurrently, with the combined mediational effects of cognitive ability (30.0%), teacher assessment (28.5%), and educational expectations (1.2%) explaining 59.8% of the total association between SES and educational attainment. Fig 1 shows the paths for Model 2d, which had excellent model fit: CFI = 1, RMSEA < .01.

**Table 6:** Standardised associations from structural equation models for mediational pathways between SES and educational attainment (N = 2,814).

		Model 2a		Model 2b		Model 2c		Model 2d	
		*b* (SE)	*p*	*b* (SE)	*p*	*b* (SE)	*p*	*b* (SE)	*p*
Formative SES	SES ← Maternal education	0.52 (0.05)	< .001	0.50 (0.05)	< .001	0.52 (0.05)	< .001	0.51 (0.05)	< .001
	SES ← Paternal education	0.63 (0.04)	< .001	0.65 (0.04)	< .001	0.64 (0.05)	< .001	0.64 (0.04)	< .001
SES → Attainment Pathways	SES → Cognitive ability	0.35 (0.02)	< .001					0.35 (0.02)	< .001
	Cognitive ability → Educational attainment	0.69 (0.02)	< .001					0.41 (0.05)	< .001
	SES → Teacher assessment			0.41 (0.02)	< .001			0.41 (0.02)	< .001
	Teacher assessment → Educational attainment			0.70 (0.02)	< .001			0.34 (0.04)	< .001
	SES → Educational expectations					0.07 (0.03)	0.004	0.07 (0.03)	0.004
	Educational expectations → Educational attainment					0.15 (0.03)	< .001	0.08 (0.03)	0.002
	SES → Educational attainment	0.24 (0.02)	< .001	0.19 (0.02)	< .001	0.47 (0.02)	< .001	0.19 (0.02)	< .001
Mediation Tests	SES → Cognition → Attainment	0.24 (0.02)	< .001					0.14 (0.02)	< .001
	SES → Teacher → Attainment			0.29 (0.02)	< .001			0.14 (0.02)	< .001
	SES → Expectations → Attainment					0.01 (0.00)	0.017	0.01 (0.00)	0.048

## Discussion

This study set out to understand whether different indicators of socioeconomic status–maternal education, paternal education, and neighbourhood SES–were related to adolescent educational attainment at age 17. Maternal education and paternal education moderately correlated, as found in other studies [92,93], yet they were independently and positively related to adolescent educational attainment. Adolescents with high-educated mothers had a significantly greater chance of higher educational attainment at age 17 than adolescents with a medium-educated mother, who themselves had a greater chance of higher educational attainment than those with low-educated mothers. The same results held for paternal education levels. We found no link between neighbourhood SES and adolescent educational attainment, nor evidence of interactions between parental education levels.

The second aim of the study was to explore whether three mediators–adolescent cognitive ability, primary school teacher assessment, and adolescent educational expectations–could explain the relation between SES (conceptualised as a composite mother education and father education score) and adolescent educational attainment. All three mediators contributed to explaining the relation between SES and educational attainment. The relations remained when all mediators were included in the same model, with over half the total relation between SES and educational attainment explained by a combination of the mediators.

### Mother education and father education both predict educational attainment

The results support a considerable body of knowledge which has found strong links between parental education and adolescent educational attainment (e.g. [19]). Adding to the literature, our findings showed that the education level of both parents matters: regardless of the education of the other parent, having a more highly educated parent, mother or father, is linked to higher adolescent educational attainment. We found no evidence of an interaction effect, suggesting that the positive relation between each parent’s education and adolescent educational attainment does not depend on the educational level of the other parent. A corollary of these results is that adolescents with neither parent having attained more than a basic education are the most likely (compared to adolescents who have at least one more highly educated parent) to themselves attain the lowest education level, perpetuating intergenerational cycles of low educational attainment. Our results emphasise the importance of both parents to their children’s educational attainment. This finding should be of interest to policy-makers and practitioners interested in reducing educational inequalities and highlights the benefits to researchers of considering the education level of both parents. Cruder measures of SES which only include the education level of the mother, the father, or the highest-educated parent, do not fully capture parental educational associations with adolescent educational attainment [24,27].

We found no relation between neighbourhood SES and adolescent educational attainment, both before and after parental SES was taken into consideration. In light of previous findings of neighbourhood effects in the Netherlands, we consider several explanations at different levels for our discrepant results. Firstly, government funding and policies may have over time reduced neighbourhood differences in the quality of learning environments [94]. Secondly, neighbourhood effects in the Netherlands may be most pronounced for adolescents from lower-SES families and those who are less academically committed, who are relatively underrepresented in the PIAMA dataset [34,35]. Thirdly, adolescents’ positive perceptions and experiences of their neighbourhood may attenuate adverse neighbourhood effects [60]. Fourthly, there may just be no neighbourhood effect in our study population. Some previous evidence of neighbourhood effects may be based on publication bias and statistical artefacts, because unmeasured variation in the composition of neighbourhood residents confounds the neighbourhood effect [95]. Due to redistributive policies concerning housing, income, and public education in the Netherlands, parental education and neighbourhood SES may not be strongly related and neighbourhoods may not be associated with individual educational outcomes [34]. Future research examining these different explanations, ideally in different regions or countries, is needed.

It is also possible that our measure of neighbourhood SES might not have fully captured the variation in neighbourhood effects. Our data did not include the full postcode of participants (i.e., only 4-digits) so we may have found weaker relations than with a more granular postcode (i.e., 6-digits), which may also explain the low correlations with parental education measures [96]. Neighbourhood effects are likely to accumulate during childhood and depend on duration of exposure, and families may move; our snapshot indicator of neighbourhood SES may be inadequate to capture such dynamic relations [97,98]. Finally, our neighbourhood indicator did not measure social embeddedness, which could be more relevant to the relation between neighbourhoods and adolescents. Social embeddedness reflects a neighbourhood’s cohesion, trust, social capital, and collective socialisation, which are related to support for educational attainment and conformity to educational norms [35,99].

### Mediation effects of cognitive ability, teacher assessment, and educational expectations

The mediation analyses provided evidence that three mechanisms explain over half of the relation between SES and adolescent educational attainment. It is striking that cognitive ability and teacher assessment both play a similar and large role, each contributing to over a quarter of the total relation when included in the same model. We expect that genetics may partly be responsible for the three mediational pathways, because cognitive ability and non-cognitive traits such as personality are substantially heritable [50]. In turn, these abilities and traits may be related to cognitive ability and the teacher assessment at age 11 and educational expectations at age 14. However, it is also important to consider other explanatory mechanisms, since there is evidence that 40–60% of variation in educational attainment cannot be explained by genetics [100]. Furthermore, there is increasing evidence that genetic influences interact with educational environments to predict educational attainment [101]. For example, the differential susceptibility model proposes that those adolescents most negatively affected by poor environments may also be most positively affected by wealthy environments [102]. Given changes between generations in average educational levels, further studies to shed light on the relative roles of genetics and the environment in such changing socioeconomic contexts could be useful [103].

These findings support theories which distinguish three types of mediational pathways linking SES and educational attainment: *primary effects* of cognitive and non-cognitive skills, *secondary effects* of educational choices and *tertiary effects* of teachers and schools [104–106]. Our results, and these theories, suggest that, teacher assessments contribute to intergenerational educational inequality (i.e., the effect of SES on educational attainment). Research has identified several possible mechanisms to explain these tertiary effects. Firstly, teachers assess adolescents’ cognitive ability, though importantly the mediation result also holds after controlling for cognitive ability. Secondly, teacher recommendations may be based on (partly genetically heritable) non-cognitive skills, such as self-control, motivation, and ability to plan, skills associated with adolescents who have higher SES parents [107]. Thirdly, teachers may have lower expectations for adolescents with low-SES parents due to, perhaps unconsciously, negatively evaluating how supportive their home environment is for their education [108]. Fourthly, low-SES parents are less able to effectively negotiate with teachers to raise their assessment levels [53,109]. In comparison, high-SES parents may use their cultural capital to influence their children’s educational trajectories by placing pressure on the teacher to change the assessment during parent‐teacher meetings [72, 110]. These mechanisms of social reproduction may explain why among children with the same cognitive ability (Cito Test) score, those with highly‐educated parents were twice as likely to end up in a higher educational track [73]. Future research could examine the role of teachers’ perception of non-cognitive skills in teacher assessment and the relative influence of these skills on later educational attainment. This would contribute to our understanding of whether these skills are important, or if self-fulling prophecies–where teacher expectations merely lead students to perform to those expectations–are at work [111].

Educational expectations also contributed to further explaining the pathway from SES to adolescent educational attainment. This result supports findings in other countries that higher educated parents encourage higher educational expectations, positive education-related behaviours, and subsequently successful educational outcomes in their children [16,63,66]. The size of the relation indicates that these educational expectations only played a small role in educational attainment relative to the other mediators. Yet the fact that educational expectations retain their mediation effect in the model with cognitive ability and teacher assessment, suggests that parents are ‘expectancy socializers’, affecting adolescent’s perceptions of their future over and above the reality of adolescent’s educational abilities [57]. Our measure of educational expectations possibly underestimated the differences in educational expectations within the sample, given that most adolescents (68%) reported the highest level of educational expectations (‘start with further or other education or study’). Future research would benefit from more fine-grained and multi-dimensional indicators of educational expectations, which reflect the complexities of adolescent future orientations and the importance of the theoretical distinction between aspirations, expectations, and plans [55,58]. This would shed light on whether Dutch adolescents are making and sticking to plans that will help them to reach their educational expectations, the compatibility of their expectations (i.e., beliefs about the future), aspirations (i.e., desires about the future) and current attainment level, and the importance they attach to educational expectations relative to other future outcomes [112].

### Strengths and limitations

Notwithstanding the strengths of this study–its longitudinal design, the inclusion of multiple mediators and the substantial number of adolescents who have contributed 17 years’ worth of data–several limitations need to be discussed. Firstly, without a genetically-informative study design it remains unknown to what extent the mediators can be explained by genetic or environmental variation in educational attainment [103]. Future studies that are able to disentangle genetic and environmental influences, explore gene-environment interactions, and include other possible mediators, such as child personality and parenting style, can enhance our understanding of the importance of the revealed mediators in the present study. Secondly, as discussed above in relation to educational expectations and neighbourhood SES, the measurement of some included variables may not have been optimal. Thirdly, to fully understand the relations and mechanisms of SES with adolescent educational attainment, research could examine the outcomes of (young) adults once they have completed their education. Later measures of attainment would show whether high-SES adolescents continue to benefit from their background later in life, perhaps through ambitious tertiary educational choices, leading to better educational, psychological, and labour market outcomes [113]. Fourthly, despite the longitudinal design of the study and timing of the measurements, which led us to hypothesise about mediational pathways from parental and neighbourhood socioeconomic status to adolescent educational attainment, our methods are unable to show that these are causal effects. The question of causality depends, among other factors, on whether there are confounders or genetic effects which explain both predictor and outcome [22,50,114]. Finally, the study was conducted on a relatively high-SES cohort of adolescents born around the turn of the century in the Netherlands. However, given the consistency of these findings with existing results, it seems likely that they are generalisable to other developed countries in a broader period.

## Conclusion

This study extends our understanding of the pathways from parental SES to adolescent educational attainment. We found that, in the Netherlands, mother education and father education levels, but not neighbourhood SES, were related to educational attainment. For the first time, we included concurrently cognitive ability, teacher assessment, and adolescent expectations as mediators of parental SES on educational attainment. All three mediators were significant, with cognitive ability and teacher assessment explaining almost 60% of the variance. Our findings show that these are important mechanisms for understanding how the socioeconomic environment in which adolescents develop is related to their educational and health trajectories.

## Supporting information

S1 Table Standardised effects of structural equation models for mediational pathways between SES (including neighbourhood SES) and educational attainment (N = 2,814). (DOCX)
