# Supplementary material for: The pathways from parental and neighbourhood socioeconomic status to adolescent educational attainment: An examination of the role of cognitive ability, teacher assessment, and educational expectations
Source: PLoS One. 2019 May 22;14(5):e0216803. doi: 10.1371/journal.pone.0216803 (PMC6530860; doi:10.1371/journal.pone.0216803)
Supplement: S1 Table — (DOCX) [file pone.0216803.s001.docx]

**Table S1. Standardised Effects of Structural Equation Models for Mediational Pathways Between SES (including Neighbourhood SES) and Educational Attainment (N = 2,814).**

|  |  | Model 2a | | Model 2b | | Model 2c | | Model 2d | |
| --- | --- | --- | --- | --- | --- | --- | --- | --- | --- |
|  |  | *b* (SE) | *p* | *b* (SE) | *p* | *b* (SE) | *p* | *b* (SE) | *p* |
| Formative SES | SES ← Maternal education | 0.52  (0.05) | < .001 | 0.50  (0.05) | < .001 | 0.51  (0.05) | < .001 | 0.51  (0.05) | < .001 |
|  | SES ← Paternal education | 0.63  (0.05) | < .001 | 0.66  (0.04) | < .001 | 0.64  (0.05) | < .001 | 0.64  (0.04) | < .001 |
|  | SES ← Neighbourhood SES | 0.01  (0.05) | 0.840 | -0.01  (0.04) | 0.833 | -0.01  (0.05) | 0.835 | 0.01  (0.04) | 0.907 |
| SES → Attainment Pathways | SES → Cognitive ability | 0.35  (0.02) | < .001 |  |  |  |  | 0.35  (0.02) | < .001 |
|  | Cognitive ability → Educational attainment | 0.69  (0.02) | < .001 |  |  |  |  | 0.42  (0.05) | < .001 |
|  | SES → Teacher assessment |  |  | 0.41  (0.02) | < .001 |  |  | 0.41  (0.02) | < .001 |
|  | Teacher assessment → Educational attainment |  |  | 0.70  (0.02) | < .001 |  |  | 0.33  (0.04) | < .001 |
|  | SES → Educational expectations |  |  |  |  | 0.07  (0.03) | 0.006 | 0.07  (0.03) | 0.006 |
|  | Educational expectations → Educational attainment |  |  |  |  | 0.15  (0.03) | < .001 | 0.08  (0.03) | 0.003 |
|  | SES → Educational attainment | 0.24  (0.02) | < .001 | 0.19  (0.02) | < .001 | 0.47  (0.02) | < .001 | 0.19  (0.02) | < .001 |
| Mediation Tests | SES → Cognition → Attainment | 0.24  (0.02) | < .001 |  |  |  |  | 0.15  (0.02) | < .001 |
|  | SES → Teacher → Attainment |  |  | 0.29  (0.02) | < .001 |  |  | 0.14  (0.02) | < .001 |
|  | SES → Expectations → Attainment |  |  |  |  | 0.01  (0.00) | 0.016 | 0.01  (0.00) | 0.047 |
